# Consumption of rice, acceptability and sensory qualities of fortified rice amongst consumers of social safety net rice in Nepal

**DOI:** 10.1371/journal.pone.0222903

**Published:** 2019-10-03

**Authors:** Anjana Rai, Macha Raja Maharjan, Helen A. Harris Fry, Parbati K. Chhetri, Purna Chandra Wasti, Naomi M. Saville

**Affiliations:** 1 Nutrition Section, UN World Food Programme Nepal, Kathmandu, Nepal; 2 Department of Population Health, London School of Hygiene & Tropical Medicine, London, England, United Kingdom; 3 Department of Food Technology and Quality Control, Ministry of Agriculture and Livestock Development, Kathmandu, Nepal; 4 Institute for Global Health, University College London, London, England, United Kingdom; CSIR-Foood Research Institute, GHANA

## Abstract

**Introduction:**

Micronutrient deficiencies are prevalent in Nepal where starchy foods constitute a large proportion of diets and consumption of micronutrient-rich foods is inadequate. We conducted a study to determine whether rice would be an appropriate vehicle for micronutrient fortification in Nepal.

**Materials and methods:**

In Bajura in remote rural Nepal, we conducted a household survey to characterize rice intakes in 195 households, and a double-blinded acceptability test with 177 social safety net rice consumers ≥18 years of age. Of these, 168 tasted fortified and unfortified rice to assess whether respondents could differentiate between fortified and non-fortified rice and their sensory properties. Rice was fortified by blending hot extruded kernels containing 6 micronutrients together with non-fortified rice at a 1:99 ratio. We used binomial tests to assess whether participants could correctly differentiate fortified rice, from non-fortified rice and paired *t*-tests to compare scores for sensory qualities of cooked fortified and non-fortified rice. We used multiple regression to test associations between per capita consumption and age, gender, wealth and food security.

**Results:**

Per capita consumption of rice (median 216g/day, IQR 144.0, 288.0) did not vary by wealth but was +52.08g, (95% CI 10.43, 93.72) higher amongst moderately to severely food insecure households compared with food secure / mildly food insecure. Most respondents could not differentiate fortified rice from non-fortified rice: 37.5% identified uncooked fortified rice and 39.3% cooked rice, which was not different from the 33% expected by chance (*p* = 0.22 and *p* = 0.09 respectively). The sensory qualities of fortified rice were acceptable (scoring 3.9 out of 5) and did not differ from non-fortified rice (*p*>0.05).

**Conclusion:**

A rice fortification programme implemented through the Nepal Food Corporation’s social safety nets has potential because purchase and consumption of rice is high and fortified rice is acceptable among consumers in remote food insecure areas of Nepal.

## Introduction

Poor diets and micronutrient deficiencies are evident across many low-income settings [1, 2]. Micronutrient deficiencies have detrimental effects across the life course, such as impaired fetal growth, low birth weight [3, 4], poor nutritional status of children [5], low productivity, intellectual growth impairment [5, 6] and poor growth and development [7, 8].

Public health programs introduced to address micronutrient deficiencies in Nepal include the vitamin A supplementation program for under-fives, iron-folic acid supplementation for pregnant and lactating women (and more recently for adolescent girls), deworming of children and pregnant women, zinc supplementation in combination with oral rehydration salts for treatment of childhood diarrhea, flour fortification at roller mills, and universal iodine fortification of salt, yet malnutrition continues to be a major driver of death and disability [9]. Prevalence of stunting, underweight and wasting among children under-five is be 36%, 27% and 10% respectively. Similarly, anemia among children and women increased over the recent years to 53% and 41% respectively [10].

One of the immediate causes of micronutrient deficiency is inadequate intake of micronutrient-rich foods [11]. Most people in Nepal consume large portions of rice in their regular diet with inadequate portions of micronutrient rich foods [1, 12–14]. Fortification of food, especially staples that are consumed by a large population, has potential to address micronutrient deficiencies [15–17]. For Nepal, rice is the most appropriate staple to fortify, as the national average per capita rice consumption is 359g/day [18] which is higher than the minimum level of consumption of 100g/day for rice fortification to be worth the investment [19–21].

Consumption of fortified rice was found to be associated with: significant reductions in anaemia and/or improved iron stores in India, Brazil [22, 23], the Philippines [24], and Mexico [25]; improved cognitive performance [26]; Vitamin A status in Cambodia [27]; reduced prevalence of anaemia and zinc deficiency in Bangladesh [28]; and, in combination with other fortification efforts, decreases in the prevalence of anaemia and folate deficiencies and neural tube defects in Costa Rica [29]. Rice fortification using hot extrusion involves the addition of essential vitamins and minerals after milling to increase its nutritional value. Fortified rice kernels are hot extruded from rice flour mixed with a micronutrient premix to look identical to rice kernels. These fortified rice kernels (FRK) are blended with non-fortified rice, usually in a ratio of 1:100 by weight, using blending technology [30]. Since this method makes rice which is difficult to distinguish from non-fortified rice, fortification offers the benefits of additional micronutrient intake without requiring consumers to change their buying, cooking, or eating habits [31].

The Nepal Food Corporation (NFC), a government-owned public enterprise, sells rice with subsidized transportation costs from 59 depots in 23 remote districts of Nepal. The areas receiving the largest amounts of NFC rice lie in the far- and mid-western hills and mountains where road access is minimal or non-existent. All households in NFC districts are eligible to purchase NFC subsidized rice from the 15000 metric tons per year social safety net rice quota [21]. A landscape analysis undertaken in Nepal suggested that introducing fortified rice into social safety nets would be the best mechanism for starting rice fortification in Nepal to enable targeting of a needy population at a reasonably small scale [21]. Experts in rice fortification recommend understanding consumer preferences and acceptability of fortified rice before initiating a rice fortification programme, which has not yet been conducted in Nepal [19, 32]. Hence, it was necessary to test the acceptability of fortified rice and per capita rice consumption in an area of Nepal where social safety net rice is regularly consumed.

This study aimed to describe the pattern of rice consumption relative to micronutrient-rich foods amongst NFC rice consumers, so as to assess the importance that fortified rice might have in the diet, and to ascertain whether they can differentiate between fortified and non-fortified rice and whether fortified rice has acceptable sensory qualities.

Ethical approval for this study was obtained from the Nepal Health Research Council ethical review board (approval number 539/2018). Written consent was taken from all participants and respondents were free to decline to answer any questions and to withdraw from tasting the rice at any time.

## Material and methods

### Study design and setting

To represent the population that would be consuming rice distributed by the Nepal Food Corporation (NFC), that may be fortified in the future, we conducted our study in the remote mountainous district of Bajura, which has the highest supply of NFC rice in the country. The small town of Kolti lies at 1300 m and is accessible by small aircraft or by walking one to two day from the district headquarters of Martadi. Due to persistent drought and limited cultivable land in this area, the population suffers from food insecurity [33]. In Bajura, rice can be grown in the lower-lying irrigated areas only, with remaining areas above 2500m in altitude producing mainly millet, wheat, barley, and buckwheat in the highest areas. The predominant ethnic groups of the lower altitude areas are Hindus of *Brahmin /Chettri*, *Thakuri* and *Dalit* castes, whilst the higher and most remote villages have ethnic Tibetan-speaking groups [34].

Our study was divided into two parts, a household survey to estimate per capita rice consumption, food insecurity status and dietary quality, and an acceptability test study to evaluate sensory qualities of fortified and unfortified rice. For the household survey, we purposively sampled clusters that were within 30 to 180 minutes walking distance of Kolti, which resulted in 13 clusters. Each cluster comprised a village except for four small villages that were close by one another, which were combined into one cluster (called “Bandhu”) due to unclear boundaries between those villages. We used population proportional to size sampling method. We sampled every fifth household and, where a respondent was not available, we sampled the next household. We interviewed household heads or other adults (≥18 years) and the person responsible for cooking, where present.

For the acceptability study, we took a convenience sample of beneficiaries of the NFC social safety net aged 18 or above who were visiting (or had recently visited) the Kolti NFC depot to purchase subsidized rice. Data were collected between 5 and 10 October 2018 during rice harvest season and just preceding the main “Dashain” festival.

### Sample size

Sample sizes were calculated using the Stata SE 15 “sampsi” command with 90% power and 0.05 two-tailed probability significance. For the household survey a sample of 181 households would provide 90% power to detect a mean that differs by a minimum of 35g from the hypothesized per capita per day consumption of rice of 307g (SD 145g) taken from the annual household survey dataset 2014–15 [18]. For the acceptability study at the Kolti NFC depot, a sample size of 173 respondents would provide 90% power to differentiate 45% of respondents correctly identifying fortified rice from the hypothesized proportion of detecting it by chance (33%) [35]. For comparison of mean 5-point hedonic scales for fortified and non-fortified rice, a sample of 177 would provide 90% power to detect 0.2 hedonic scale points difference from a mean of 3.68 and standard deviation (SD) of 0.82, using means and SDs from a previous hedonic scale for food preference applied elsewhere in Nepal [36, 37]. So, we took 177 as our target sample size for the acceptability study.

### Data collection

Interviewers, who received five days of training, administered questionnaires using CommCare version 2.44.3 data collection platform for android smartphones (http://www.commcarehq.org/home/). During the acceptability study at the NFC depot, household-related socio-economic questions were asked in one outdoor location, while all rice-tasting tests were conducted in three rice tasting booths inside tents with one respondent at a time, so that participants could taste rice samples and respond unobserved by others. Respondents were given unique barcode ID cards, which were scanned by smartphones, to enable respondent questionnaire and taste testing responses to be linked. The household survey and acceptability study sought similar information on rice consumption and diet (using the same questionnaire) but additional triangle and hedonic tests of rice were added to the acceptability study.

### Measurement of variables

In both acceptability study and the household survey, we estimated individual rice consumption by measuring respondents’ self-reported intakes of cooked rice the last time they ate rice using a life-sized photographic atlas of portions of different weights of cooked white rice, which has been created and validated in Nepal [38]. We converted these estimated boiled rice quantities to uncooked rice by multiplying the weight of cooked rice by 0.36 [39]. Then, we multiplied the resulting weight of rice by number of times it was typically consumed per day.

To get an overall household consumption estimate, we also asked the cook in the household survey and respondent in the acceptability study to measure the amount of uncooked rice prepared for the whole household last time they had a rice meal and weighed this on electronic kitchen scales (Salter, UK). Per capita consumption of rice by household members was estimated by multiplying the weight of dry rice by number of times rice was cooked per day and dividing the resulting weight by number of household members regardless of age. To gain an estimate of the consumption of subsidized rice only (i.e. the rice that will be fortified in the rice fortification programme), we multiplied the typical amount of subsidized rice consumed per month, by the number of months subsidized rice was purchased, divided it by 365 days in the year, and again divided this by the number of persons per household to get an average amount consumed per capita per day over the year. Consumption of other rice products such as beaten- and puffed- rice were not estimated, since these are not likely to be fortifiable and are also consumed relatively rarely in this area of Nepal.

We questioned respondents on household food insecurity using the Household Food Insecurity Access Scale (HFIAS) [40], and months of adequate household food provisioning (MAHFP) [41]. To estimate dietary quality of the household we asked the respondent to recall consumption of the 10 food groups defined in the minimum dietary diversity score for women (MDD-W) in the last 24 hours and 7 days (namely cereals/root tubers, pulses, nuts/seeds, dairy, meat/fish, eggs, green leafy vegetables, vitamin A-rich fruits and vegetables, other vegetables, other fruits). We defined consumption of five or more food groups over the recall period as ‘adequate’ [42]. Despite the fact that the MDD-W score was designed to look at consumption by women rather than households, we chose this score over other alternatives available because it focuses upon micronutrient-rich foods and excludes oils/fats, sugars, and salt/spices/tea/coffee which are included in the household dietary diversity score [43].

To generate estimates which could be compared with other WFP working areas, we also calculated food consumption scores as used in WFP food security monitoring [44, 45]. This asks respondents to recall consumption of nine food groups (main staples including cereals and starchy tubers, pulses/nuts, vegetables, fruits, meat/fish/eggs, dairy, sugar, oils/fats, condiments) and the number of days in a week that these food groups were consumed. Different food groups are assigned different weights depending on their nutritional values (i.e. sugar and fat are weighted 0.5 while other groups have a weight of 1). The consumption frequencies for each of the food groups were summed followed by multiplying the frequency of each food group by its weight. The food group scores were then summed to create the food consumption score for each household. Instead of the general food consumption score categories, we used a cutoff that adjusts for consumption of oils and sugar in the past week [45] and categorized consumption scores into poor (0–28), borderline (>28–42) and acceptable (>42). We constructed a wealth index on ownership of assets, education, toilet, fuel, electricity and number of rooms using principal component analysis and the distribution was divided into tertiles.

In the acceptability study, we conducted triangle- and hedonic- sensory tests [46] on Nepal Food Corporation *Sona Mansuli* variety of polished white rice which was fortified with hot-extruded fortified rice kernels (FRK) received from WFP Bangladesh (supplied by Igloo Foods Ltd., Dhaka, Bangladesh). The fortified rice kernels were fortified with six micronutrients as per the Bangladesh fortified rice standard. The levels cited here are the ranges expected at the household level after losses during storage: Vitamin A (palmitate) 150–215 *μg*, Vitamin B_1_ (Thiamine mononitrate) 0.4–0.6 mg, Vitamin B_12_ (cyanocobalamine) 1.00–1.45 *μg*, Vitamin B_9_ (folic acid) 130–190 *μg*, Iron (ferric pyrophoshate) 5–7 mg, and Zinc (zinc oxide) 3.5–4.5 mg. These kernels were blended in a ratio of 1:99 FRK to raw rice by weight using a hand operated blender (HOB) comprising a stainless-steel inclined barrel rotated with a handle, previously used by ‘Chhimeki Sanstha’ in a small-scale blended complementary food fortification project. A total of 12kg of fortified rice was produced.

Before producing the fortified rice, we tested the effectiveness of blending to ensure a 1:99 ratio by blending rice grains dyed with orange food colour (used as a proxy for FRK) together with the rice to be used in the test. These were mixed at 1% rate by rotating the HOB 100 times by hand. After blending, samples of the rice were weighed into portions of 100g. Then we separated the kernels into dyed and non-coloured kernels and weighed them to establish the ratio in the sample. The average concentration of coloured rice over five test runs was 0.93%, which varies by 7% from the target concentration of 1.0%, which is well within the acceptable variation of ±15%. The coefficient of variation was 5%, which is well within the acceptable limit of up to 14% so the HOB was considered suitable for producing fortified rice for the acceptability test.

In the triangle test (as used by others [35, 47]), respondents were asked to differentiate the odd one out of 3 small paper cups of rice. One of the cup contained fortified rice and two non-fortified rice and respondents were simply asked to say which one was different from the others and to guess which was different if they thought they all looked or tasted the same. This triangle test was applied for uncooked rice where respondents visually inspected the rice, and for cooked rice where they tasted all 3 bowls of rice to determine which bowl of rice was different from the other two. Each bowl contained ~35g of cooked rice. For uncooked rice, the paper cups were filled to three-quarters full.

In the hedonic test, respondents were asked to evaluate five sensory qualities of cooked rice by tasting rice from a paper cup and ranking its taste, smell, colour, texture, appearance, as well as overall liking. We performed this for cooked fortified and non-fortified rice using a 5-point Likert scale which ranged from very bad (1) to very good (5) for each sensory quality. These hedonic scales were indicated with emoji (smiley face) images to enable illiterate respondents to interpret the scale. All bowls of rice in the triangle and hedonic tests were presented to look identical with similar serving size. Bowls were only differentiated by unique identifying QR coded stickers which were meaningless to any observer but identified whether the rice was fortified or not once scanned by a mobile phone and decoded by a data analyst after completion of the survey. The bowls were placed in pre-generated random arrangement. In both tests, respondents were asked to take a sip of water before tasting each separate portion of rice to clean the palate. The tests were double-blinded: respondents and interviewers did not know which rice was fortified rice, and which was not. Only the lead field researcher (AR), who served the rice, knew which rice was which. The triangle and hedonic tests were piloted thoroughly at the Kathmandu WFP office before conducting the real tests in Kolti, Bajura.

### Statistical analysis

In the household survey, we used the svyset function with weighting to account for the weighted cluster survey sampling design. We calculated descriptive statistics for socio-economic characteristics of respondents, dietary diversity, food security, purchase and consumption of rice. We used multivariable linear regression to assess associations between age, gender, wealth and food security and per capita consumption of rice.

For triangle tests, we used a binomial test to test whether the proportion of participants correctly distinguishing between fortified rice and non-fortified rice differed from the 0.33 who would choose the correct bowl by chance. We used paired *t*-tests for hedonic test responses to determine whether scores differed by taste, smell, colour, texture, appearance and overall liking for cooked fortified rice and non-fortified rice. All analyses were performed using Stata/IC 15.1.

## Results

The response rate for the household survey was 100%. In the acceptability study, we had a non-response rate of 16.4%. Most of the non-response was because they did not want to taste the rice for triangle and hedonic tests because of religious taboos forbidding them from eating rice cooked by non-family members. The analysis for the acceptability study includes 168 respondents who completed both socio-economic questions and tasted rice in triangle and hedonic tests.

Table 1 presents the socio-economic characteristics of acceptability study and household survey respondents. Respondents were older in the household survey than the acceptability survey; the median age of respondents was 45.0 years and 27.5 years respectively. There were more male respondents in the household survey (65.4%) than acceptability study (57.1%). Most respondents were hill *Brahmin/Chhetri* (64%) followed by hill *Dalits*. Most households had some livestock, with the majority owning cattle, buffaloes, and sheep or goats.

**Table 1:** Socio-economic characteristics of respondents in household and acceptability surveys.

		Household Survey (*N* = 195)	Acceptability study (*N* = 168)
Variables	Categories	Median (IQR)	Median (IQR)
Age (years)		45.0 (30.0, 60.0)	27.5 (20.0, 35.5)
		**% (n)**	**% (n)**
Gender	Women	34.6 (67)	42.9 (72)
	Men	65.4 (128)	57.1 (96)
Caste group	Hill *Brahman/Chhetri*	63.5 (124)	63.7 (107)
	Hill *Dalit*	16.3 (32)	22.0 (37)
	*Thakuri*	8.9 (17)	11.9 (20)
	Hill *Janjati*	8.6 (17)	1.8 (3)
	Muslim	2.7 (5)	0.0 (0)
	Others (*Sanyashi*, *Dasnami*)	0.0 (0)	0.6 (1)
Education	Never went to school	41.8 (82)	26.2 (44)
	Primary to lower secondary	38.6 (75)	40.5 (68)
	Secondary and above	19.6 (38)	33.3 (56)
Religion	Hindu	88.7 (173)	98.8 (166)
	Muslim	2.7 (5)	0 (0)
	Buddhist	8.6 (17)	1.2 (2)
Household size	0–5	34.2 (67)	32.1 (54)
	6–10	54.4 (106)	61.9 (104)
	> = 11	11.4 (22)	6.0 (10)
Ownership of toilet	No	5.4 (11)	3.6 (6)
	Yes	94.6 (184)	96.4 (162)
Wealth tertiles	Lower	33.1 (65)	33.3 (56)
	Middle	33.6 (65)	33.3 (56)
	Higher	33.3 (65)	33.3 (56)
Ownership of livestock	Yes	94.2 (184)	91.1 (153)
	No	5.8 (11)	8.9 (15)
	Has cattle	91.6 (179)	85.1 (143)
	Has buffaloes	28.4 (56)	47.6 (80)
	Has sheep/goats	50.9 (99)	47.0 (79)
Food security as measured by the Household Food Insecurity Access Scale (HFIAS)	Food secure to mildly insecure	35.5 (69)	41.7 (70)
	Moderate to severe	64.5 (126)	58.3 (98)
Food consumption score (FCS)	Poor (0–28)	14.0 (28)	9.5 (16)
	Borderline (28.5–42)	27.4 (53)	32.7 (55)
	Acceptable (>42.5)	58.6 (114)	57.7 (97)
Purchases subsidized rice		83.5 (163)	100.0 (168)
Purchases non-subsidized rice		74.9 (146)	42.9 (72)
Indicators using food groups out of the 10 in the Dietary Diversity for Woman score (MDD-W)			
Adequacy of MDD-W Food groups consumed by the household in last 24h	Inadequate <5 groups	79.3 (155)	80.4 (135)
	Adequate > = 5 groups	20.7 (40)	19.6 (33)
Adequacy of MDD-W Food groups consumed by the household in last 7days	Inadequate <5 groups	85.8 (167)	79.8 (134)
	Adequate > = <5 groups	14.2 (28)	20.2 (34)
Mean (SD) MDD-W Food groups consumed by the household in last 24h		3.3 (1.4)	3.5 (1.6)
Mean (SD) MDD-W Food groups consumed by the household in last 7 days		2.6 (1.5)	3.0 (1.7)

Only one fifth of households consumed five out of the 10 food groups used in the MDD-W Score, in the last 24 hours. Slightly more households in the household survey (64.5%) were moderately to severely food insecure than in the acceptability study (58.3%). Nearly 40% of households had borderline or poor seven-day food consumption scores. All respondents in the acceptability study and 84% in the household survey purchased subsidized rice and 42.9% and 74.9% of households purchased nonsubsidized rice in the acceptability study and household survey respectively.

Table 2 shows the respondents’ individual (using food atlas) and household (using weighed dry rice) per capita consumption of rice and associations by age, gender, wealth and food security status. Respondent’s median per capita consumption using the food atlas was 216.0g (IQR 144.0, 288.0) per day for both acceptability study and household survey respondents. The mean per capita consumption was 221.8g (104.8) and 220.4g (135.6) per day for acceptability study and household survey respectively. Household’s per capita consumption using weighed uncooked rice was 223.4g (IQR 155.6, 277.9) and 215.5g (IQR 160.2, 284.5) per day for the household survey and acceptability study respectively. Median annual household rice consumption was 540 kg (390, 920) and 469 kg (360, 720) in acceptability study and household survey respectively.

**Table 2:** Individual and household per capita consumption of rice and association of age, gender, wealth and food security with individual per capita consumption.

	Rice consumption (g/day) in household survey (*N* = 195)				Rice consumption (g/day) in acceptability study (*N* = 168)			
Variables	Mean (SD)	Median (IQR)	*N*	Adjusted coefficient (CI)	Mean (SD)	Median (IQR)	*N*	Adjusted coefficient (CI)
**Individual per capita consumption**^1^ (g/day)	221.8 (104.8)	216.0 (144.0, 288.0)	195		220.4 (135.6)	216.0 (144.0, 288.0)	168	
**Age**								
18–29	242.0 (100.0)	216.0 (144.0, 288.0)	43	**Ref.**	213.0 (132.4)	144.0 (144.0, 216.0)	95	**Ref.**
30–44	244.2 (112.0)	216.0 (144.0,288.0)	50	-9.54 (-60.95, 41.87)	224.6 (135.5)	216.0 (144.0, 288.0)	48	1.87 (-45.91, 49.65)
45–80	201.9 (100.1)	144.0 (144.0,288.0)	102	-63.74 (-97.86, -29.63)**	240.5 (150.4)	216.0 (144.0, 288.0)	25	22.71 (-38.16, 83.57)
**Gender of respondent**								
Men	234.8 (112.6)	216.0 (144.0, 288.0)	128	**Ref.**	237.2 (147.5)	216.0 (144.0, 288.0)	96	**Ref.**
Women	197.0 (83.3)	216.0 (144.0, 216.0)	67	-51.31 (-81.59, -21.04)**	198.0 (115.0)	144.0 (144.0, 216.0)	72	-38.33 (-80.06, 3.41)
**Household asset tertiles**								
Lower	226.2 (106.0)	216.0 (144.0, 288.0)	65	**Ref.**	235.9 (161.8)	216.0 (144.0, 288.0)	56	**Ref.**
Middle	220.2 (110.0)	216.0 (144.0, 288.0)	65	-14.93 (-77.60, 47.73)	215.7 (106.2)	216.0 (144.0, 288.0)	56	-15.83 (-66.49, 34.83)
Higher	219.0 (99.6)	216.0 (144.0, 288.0)	65	-22.44 (-56.22, 11.34)	209.6 (134.0)	216.0 (144.0, 216.0)	56	-17.25 (-68.72, 34.22)
**Household food security**								
Food secure to mildly insecure	208.2 (85.2)	216.0 (144.0, 288.0)	69	**Ref.**	190.8 (84.7)	216.0 (144.0, 216.0)	70	**Ref.**
Moderate to severely food insecure	229.3 (113.8)	216.0 (144.0, 288.0)	126	15.40 (-20.61, 51.41)	241.5 (159.6)	216.0 (144.0, 288.0)	98	52.08 (10.43, 93.72)*
***Using weighed dry rice***								
Household per capita consumption^2^ (g/day)	228.0 (138.6)	223.4 (155.6, 277.9)	195		236.8 (153.7)	215.5 (160.2, 284.5)	168	

*p<0.05,

**p<0.01

^1^ Individual per capita consumption estimated from photographic food atlas of rice portion sizes. Note that since the estimates are based on 6 portion size photos rather than a continuous measure of rice consumption, median values may look identical across categories.

^2^ Household per capita consumption estimated from weighed dry rice cooked for the household

In the household survey, respondents aged 45–80 consumed -63.74g (95% CI -97.86, -29.63) less rice compared to the 18–29 age group and women also consumed -51.31g (CI -81.59, -21.04) less rice than men, but differences by wealth and food security were not significant. In the acceptability study, there were no significant differences in individual rice consumption by age, gender or wealth but respondents from moderately to severely food insecure households consumed 52.08 g (CI 10.43, 93.72) more rice than respondents from food secure to mildly insecure households.

Table 3 shows findings on land ownership, and production and purchase of rice from the two surveys and an estimate of consumption of subsidized rice only as this would be the fortified rice in a rice fortification programme. Most respondents owned land, though landholdings were small. Most (~80%) cultivated some rice, but yields in 2017 were moderate (120 to 150 kg/ year) and were only enough to feed the household for an average of three months, so households depend on rice purchase for most of the year. Households purchased 30 kg subsidized rice and 40 kg of nonsubsidized rice in the preceding month in both samples. However, a higher price (NPR 1650 vs 1320) for subsidized rice was reported by respondents of the household survey as compared to the acceptability study households, perhaps because they bought different varieties of NFC rice or paid for transport of the rice. Most (>91%) households cooked rice once per day (median times that rice was cooked per week 7, IQR: 7,8) and families tend to consume all that they cook at one mealtime. In a typical month, households purchased 30 kg of subsidized and 40 kg of non-subsidized rice in both the studies. In the last year, households in the household survey purchased subsidized rice for six months compared to nine months in the acceptability study. Households in both studies purchased non-subsidized rice for three months. Compared with acceptability study respondents, household survey respondents had relatively higher production of rice in 2017 and more months of adequate household food provisioning but nevertheless both samples experienced 5 to 6 months of inadequate household food provisioning. Assuming that households consumed all the rice purchased and spreading the consumption of subsidized rice over the whole year, even though it is purchased for 6 to 9 months of the year old, median per capita consumption of subsidized rice only (i.e. the rice that would be fortified in future) was 123.3 (IQR 82.1, 199.1) in the acceptability study and 117.4 (IQR 65.8, 164.4).

**Table 3:** Ownership of land, production and purchase of rice.

	Household Survey (*N* = 195)		Acceptability study (*N* = 168)	
Variables	%	*n*	%	*n*
Ownership of land (yes)	96.9	189	95.2	160
Cultivated land for rice (yes)	80.1	156	81.6	137
Rice consumption only once per day	93.1	182	91.1	153
	**Median (IQR)**	***n***	**Median (IQR)**	***n***
Ownership of land (hectares)	0.20 (0.08,0.41)	189	0.24 (0.12,0.50)	160
Rice yield in 2017 (kg)	150 (60,390)	155	120 (60,300)	137
Consumed home-produced rice (months)	3 (2,4)	155	3 (1,4)	137
Number of months of adequate household food provisioning	7 (5,9)	195	6 (3,9)	168
Number of months subsidized rice was purchased in last 12 months	6 (4,9)	163	9 (6,11)	168
Subsidized rice purchased in a typical month (kg)	30 (30, 40) 40.1 (19.4)*	163	30 (30, 40) 39.8 (20.3)*	168
Subsidized rice purchased in the preceding month of September 2018, (kg)	30 (30,60)	163	30 (30,55)	168
Per capita consumption of subsidized rice (that will be fortified in the rice fortification programme) calculated from purchased quantity (g/day)	117.4 (65.8, 164.4) *133*.*2 (102*.7)*	163	123.3 (82.1, 199.1) *152*.*8 (109*.1)*	168
Number of months non-subsidized rice was purchased in last 12 months	3 (2, 7) 5.0 (3.5)*	142	3 (2, 4) 3.2 (1.6)*	68
Non- subsidized rice purchased in a typical month, kg	40 (40, 50) *50*.*2 (29*.5)*	142	40 (30, 40) *38*.*4 (15*.0)*	68
Nonsubsidized rice purchased last month (September 2018), kg	40 (30,50)	142	40 (30,40)	68
Money spent last month on subsidized rice (NPR)	1650 (1300, 2330)	163	1320 (980, 2000)	168
Money spent last month on non-subsidized rice (NPR)	2500 (1900, 3000)	142	2500 (2200, 3000)	68
Frequency of rice cooked per week in household	7 (7,7)	195	7 (7,8)	168

*Mean (SD)

**Fig 1:**
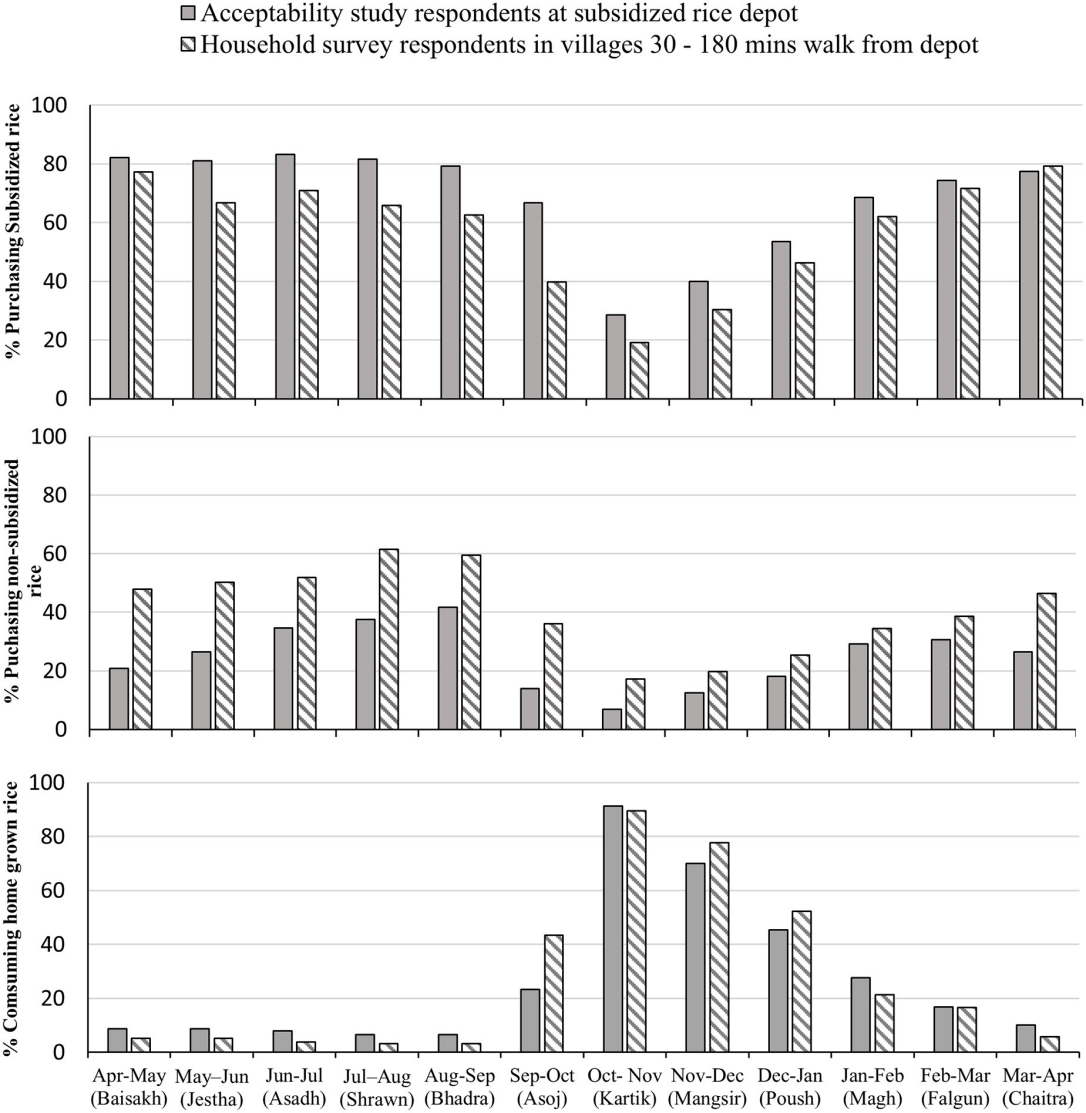
Percentage of households in the acceptability study and household survey that purchased subsidized and nonsubsidized rice in each Nepalese month of the year.

**Fig 2:**
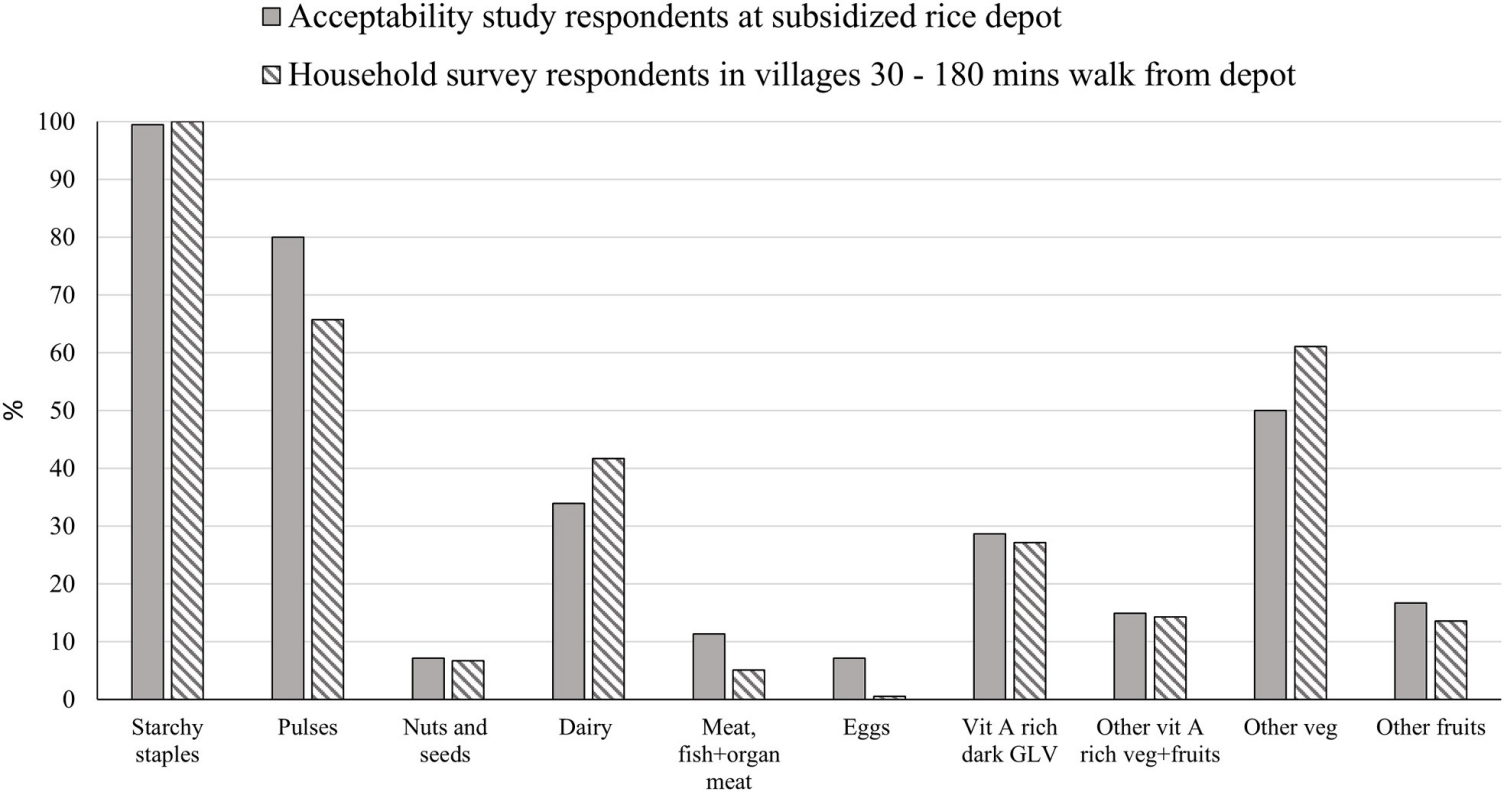
Percentage of households that consumed each of ten food groups in the last 24 hours in the acceptability study and household survey.

Seasonality of subsidized and non-subsidized rice purchase is given in Fig 1. Most households purchased both subsidized and non-subsidized rice throughout the year with decreases between October and December when the festivals are celebrated, and rice is harvested. Consumption of home-grown rice peaked at the time of harvest (Oct/Nov, showing an inverse pattern to that of rice purchase. Household consumption of 10 food groups (as used in the MDD-W) in the last 24 hours in the acceptability and household surveys is given in Fig 2. All households consumed cereals, and most consumed pulses (≥65%) and other vegetables (≥50%). Other food groups consumed by more than 10% of households (in order of decreasing prevalence) were dairy, green leafy vegetables, vitamin A rich vegetables / fruits and other fruits. Consumption of nuts/seeds, meat/fish, and eggs was minimal.

Results of the triangle test (Table 4) show that 100% of the participants said that one of the bowl of rice was different than the other two in both raw and cooked tests while 37.5% and 39.3% of participants respectively were able to correctly differentiate raw and cooked fortified rice from non-fortified rice. This was not significantly different from the hypothesised 33.3% expected by chance. Mean hedonic scale scores (Table 5) were acceptable for fortified rice, exceeding 3.5 out of 5 for taste, smell, texture, colour, appearance and overall liking. The mean and median scores for fortified rice were almost identical to those for non-fortified rice.

**Table 4:** Binomial test of proportions for triangle test (*N* = 168).

Triangle test	Assumed percentage of respondents identifying fortified rice	Observed percentage of respondents identifying fortified rice	Respondents who identified fortified rice	Two-sided test p-value
Raw rice	33.3%	37.5	63	0.22
Cooked rice	33.3%	39.3	66	0.09

**Table 5:** Qualities for fortified and non-fortified rice (*N* = 168).

Type of Rice	Taste	Smell	Texture	Colour	Appearance	Overall
Fortified rice (Mean, SD)	3.6 (0.8)	3.6 (0.9)	3.6 (0.8)	3.7 (0.8)	3.9 (0.7)	3.9 (0.8)
Non-fortified rice (Mean, SD)	3.5 (0.9)	3.6 (1.0)	3.6 (0.8)	3.7 (0.8)	3.8 (0.8)	3.9 (0.9)
t-statistic for paired *t*-test	1.50	-0.12	0.00	0.67	0.91	0.57
*p* value for paired *t*-test	0.14	0.90	1.00	0.51	0.37	0.57

## Discussion

Our study shows that in a remote population with high food insecurity, low levels of dietary diversity and low intake of micronutrient-rich foods where rice is consumed every day, rice fortified with micronutrients is highly acceptable. Although this population mostly consumes rice only once per day, the per capita consumption exceeds 200g per day. Since most studies which have proved efficacy of rice fortification have tested intakes of 70 to 100g of cooked fortified rice per day [16] we deem these consumption levels to be high enough for rice fortification to be effective. The sensory qualities of fortified rice did not differ from non-fortified rice and the majority of the respondents were not able to differentiate fortified rice from non-fortified rice. Even though most households owned small landholdings and cultivated rice, production was low and was only enough for an average of three months’ supply, so most depend on subsidized rice provided by the Nepal Food Corporation. Averaged across the year consumption of subsidized rice alone (that would be fortified rice in the fortification programme) was around 120g per capita per day. Hence, fortifying subsidized rice with micronutrients has potential as an approach to improve nutrient intake in the areas where subsidized rice is distributed.

Our findings on consumption of food groups in the last 24 hours are similar to the findings of other studies from Nepal [48, 49] and other Asian countries [50]. Starchy staples were consumed by all, at least two third of the households consumed legumes and at least 50% consumed some vegetables but intakes of vitamin A-rich vegetables and fruits, dairy, fruits, meat, fish, eggs and nuts / seeds were inadequate. The frequency of rice consumption per week was also low for our population compared to studies from the plains and mid-hills of Nepal where people consume rice twice per day on average [13, 14, 48]. This may be because the mountain district of Bajura has relatively low production of rice because much of the land is above the rice growing altitude, or on rain-fed terraces unsuitable for irrigation. The area is drought-prone and is more suited to crops such as millet, wheat, barley and buckwheat, which form the staple in the meals that rice is not consumed [33]. Household dietary intake and food security, as measured by the MDD-W and HFIAS respectively, indicated poor dietary quality with <21% households consuming the minimum 5 food groups and indicated high levels of food insecurity (>58%). However, the percentage with an acceptable food consumption score (FCS) over 7 days was 58%. It would appear that despite weighting to reduce the influence of fats and sugars, that the FCS is not adequately capturing adequacy in terms of dietary diversity and hence may not reliably estimate nutritional quality.

We found a small difference in the percentage of households buying subsidized and non-subsidized rice between the acceptability study, which sampled people who came to the depot to purchase subsidized rice, and the household survey, which was a random sample of households outside of the small town of Kolti. Compared to the acceptability study, fewer households in the household survey bought subsidized rice and relatively more households bought non-subsidized rice. This could be because the remoter high-altitude locations rely on their nearest markets where non-subsidized rice is available. It is also notable that the timing of our study was at the time when rice purchase was lowest in the year (Fig 2) so our purchase estimates will be conservative compared to other seasons and we may expect higher amounts of rice purchased during other seasons.

The finding that older respondents consume significantly less rice than younger respondents may be because of lower energy requirements of older respondents. Women in the household survey also consumed significantly less rice than men, which is consistent with several studies from plains of Nepal [12, 49, 51]. This may be due to unequal intra-household food allocation, as was found in the plains using the same food atlas [49] or possibly lower energy requirements of women or substitution of rice for another locally available cereal by women. We speculate that respondents from moderately to severely food insecure households have poor access to other food groups compared to food secure and mildly insecure households and therefore their meals constitute relatively larger portions of rice.

We found no difference in hedonic scores for sensory qualities of nonfortified rice and rice blended with fortified rice kernels in a ratio to 1:99 by weight. Most respondents were unable to distinguish fortified from unfortified rice, whether as uncooked rice or when cooked. This means that fortified rice is likely to be acceptable in this setting and that the main determinants of acceptability of fortified rice are palatability of the type of rice that is fortified, and perceptions around fortification. A study from India similarly reported no difference in mean hedonic scores for rice fortified with ferric pyrophosphate and nonfortified rice [52]. A study in Bangladesh among social safety net rice consumers also found that the smell, colour and taste of fortified rice was acceptable to majority of the participants [53]. Moretti et al. (2005) used a triangle test for sensory comparison of rice fortified with ferric pyrophosphate and nonfortified rice involving 18 panelists and reported that fortified rice is acceptable and resembles nonfortified rice in both cooked and uncooked form [54]. Another study that tested for sensory qualities found that fortified rice was liked by panelists using a seven-point rating scale. Of the 14 participants, nine detected some difference in taste, texture and colour of fortified rice [55]. Moretti et al. (2006), in another study used a triangle test to determine whether 24 women could distinguish rice fortified with iron from non-fortified rice and found that rice fortified with both 3 and 5 mg of iron was indistinguishable from nonfortified rice both in uncooked and cooked forms [47]. One study in Cambodia and Vietnam had contrasting results to ours: fortified rice scored higher in hedonic tests for smell and taste compared to non-fortified rice and most respondents were able to differentiate fortified rice from unfortified rice [35].

Since our study population consumes social safety net rice and found fortified rice acceptable, our findings confirm that rice is an appropriate vehicle for micronutrient fortification and can reach remote populations, especially since consumption of cereals was found to be higher than any other food group and rice is eaten daily. Policy makers and programme planners should focus their resources in implementing a rice fortification programme in Nepal, to see improvements in population level nutritional status especially in remote areas. While most of the households had some cultivable land and owned livestock, food insecurity was high and their intake of meat, eggs and vegetables was still poor. Therefore, programmes that also support poultry and livestock husbandry, kitchen gardening and improved irrigation in dry, drought prone areas may be important in reducing food security and improving diet quality and income.

Further research could explore perceptions about rice fortification, acceptability among wider populations beyond social safety nets, willingness to pay for fortified rice in the wider Nepali population and likelihood of choosing one product over another. Experience with iodised salt in Nepal is that fortification has not affected acceptability, so we hypothesise that negative perceptions about fortification are likely to be less of a barrier to consumption of fortified rice than marketing and cost issues. Efforts at mandatory fortification of wheat flour in Nepal have yet to be assessed systematically. A study investigating enabling and hindering factors that influence wheat flour fortification efforts might help to inform the rice fortification process in Nepal.

Limitations of our study include that we used convenience sampling for the acceptability study by selecting all NFC recipients over 18 years who reported that they purchased subsidized rice on the study days, however we reduced bias by sampling everyone meeting our inclusion criteria. The findings of the acceptability tests also may not be generalizable to populations who do not consume social safety net rice but populations that do not consume NFC rice are likely to be better off and may have better diets. This study was conducted immediately before the national festival “*Dashain*” and during rice harvest season, which may have influenced the number of people visiting the Kolti NFC depot and the amounts of rice purchased. We estimated rice consumption by assuming that the same amount was consumed at each rice meal (if people ate rice more than once per day), and that all the rice was consumed in one meal. Whilst this may have led to some inaccuracy, this assumption was based upon our knowledge of the local context. People tend to eat two meals with large servings of rice or another staple such as wheat, barley, millet or buckwheat per day and consume all the food in one sitting. Snacking is less common and reheating of leftovers not very commonly practiced, so our estimation of household consumption from weighing the dry rice cooked is likely to be indicative of the amount consumed that day, especially as most families ate rice only once per day. Our findings on diet, rice purchase and consumption patterns may be generalizable to other social safety net rice distribution areas but are not representative of national population patterns.

## Conclusions

Consumption of all rice and of social safety net subsidized rice that will be fortified under the rice fortification programme is sufficiently high to consider rice as a potential vehicle for fortification with micronutrients. Fortified rice is acceptable to consumers in a remote area where social safety net rice is frequently consumed. Based on this we conclude that the Rice Fortification Programme can be implemented initially with Nepal Food Corporation’s Subsidized Rice Supply Programme in order to improve the micronutrient status of vulnerable populations in remote areas of Nepal.

## Abbreviations

FCS: Food Consumption Score
FKR: Fortified Rice Kernels
HFIAS: Household Food Insecurity Access Scale
HOB: Hand Operated Blender
IQR: Inter-quartile Range
MDD-W: Minimum Dietary Diversity Score for Women
MAHFP: Months of Adequate Household Food Provisioning
NFC: Nepal Food Corporation
NPR: Nepalese Rupee
QR code: quick response code
SD: Standard Deviation
WFP: World Food Programme
