# Correction: Consumption of rice, acceptability and sensory qualities of fortified rice amongst consumers of social safety net rice in Nepal

**DOI:** 10.1371/journal.pone.0227112

**Published:** 2019-12-19

**Authors:** Anjana Rai, Macha Raja Maharjan, Helen A. Harris Fry, Parbati K. Chhetri, Purna Chandra Wasti, Naomi M. Saville

There are errors in the Funding statement. The publisher apologizes for the errors. The correct Funding statement is as follows: This study was funded by United Nations World Food Programme, Nepal. The funder provided support in the form of salaries for authors AR, MRM, PKC and NMS who designed and implemented the study. The specific roles of these authors are articulated in the ‘author contributions’ section. Aside from funding salaries and paying implementation costs, United Nations World Food Programme did not have any additional role in the study design, data collection and analysis, decision to publish, or preparation of the manuscript. Partial funding of author HHF during the write-up period was funded by a Sir Henry Wellcome Fellowship. Grant number: 210894/Z/18/Z.

The ORCID iD is missing for the third author. Author Helen A. Harris Fry’s ORCID iD is: 0000-0003-2367-908X (https://orcid.org/0000-0003-2367-908X).

Due to typographical errors in the underlying data, there are errors throughout the article in the reporting of the food consumption score (FCS), the percentage of households consuming adequate Minimum Dietary Diversity of Women (MDD-W) food groups in the last seven days, and the mean score for the households consuming Minimum Dietary Diversity of Women (MDDW) food groups for seven days. The correct percentage of households with an acceptable food consumption score (FCS) over 7 days is 61%. These errors are present in Table 1. Please see the correct Table 1 here.

**Table 1:** Socio-economic characteristics of respondents in household and acceptability surveys.

		Household Survey (*N* = 195)	Acceptability study (*N* = 168)
Variables	Categories	Median (IQR)	Median (IQR)
Age (years)		45.0 (30.0, 60.0)	27.5 (20.0, 35.5)
	** **	**% (n)**	**% (n)**
Gender	Women	34.6 (67)	42.9 (72)
	Men	65.4 (128)	57.1 (96)
Caste group	Hill *Brahman/Chhetri*	63.5 (124)	63.7 (107)
	Hill *Dalit*	16.3 (32)	22.0 (37)
	*Thakuri*	8.9 (17)	11.9 (20)
	Hill *Janjati*	8.6 (17)	1.8 (3)
	Muslim	2.7 (5)	0.0 (0)
	Others (*Sanyashi*, *Dasnami*)	0.0 (0)	0.6 (1)
Education	Never went to school	41.8 (82)	26.2 (44)
	Primary to lower secondary	38.6 (75)	40.5 (68)
	Secondary and above	19.6 (38)	33.3 (56)
Religion	Hindu	88.7 (173)	98.8 (166)
	Muslim	2.7 (5)	0 (0)
	Buddhist	8.6 (17)	1.2 (2)
Household size	0–5	34.2 (67)	32.1 (54)
	6–10	54.4 (106)	61.9 (104)
	> = 11	11.4 (22)	6.0 (10)
Ownership of toilet	No	5.4 (11)	3.6 (6)
	Yes	94.6 (184)	96.4 (162)
Wealth tertiles	Lower	33.1 (65)	33.3 (56)
	Middle	33.6 (65)	33.3 (56)
	Higher	33.3 (65)	33.3 (56)
Ownership of livestock	Yes	94.2 (184)	91.1 (153)
	No	5.8 (11)	8.9 (15)
	Has cattle	91.6 (179)	85.1 (143)
	Has buffaloes	28.4 (56)	47.6 (80)
	Has sheep/goats	50.9 (99)	47.0 (79)
Food security as measured by the Household Food Insecurity Access Scale (HFIAS)	Food secure to mildly insecure	35.5 (69)	41.7 (70)
	Moderate to severe	64.5 (126)	58.3 (98)
Food consumption score (FCS)	Poor (0–28)	13.5 (27)	9.5 (16)
	Borderline (28.5–42)	25.8 (50)	30.4 (51)
	Acceptable (>42.5)	60.6 (118)	60.1 (101)
Purchases subsidized rice		83.5 (163)	100.0 (168)
Purchases non-subsidized rice		74.9 (146)	42.9 (72)
Indicators using food groups out of the 10 in the Dietary Diversity for Woman score (MDD-W)			
Adequacy of MDD-W Food groups consumed by the household in last 24h	Inadequate <5 groups	79.3 (155)	80.4 (135)
	Adequate > = 5 groups	20.7 (40)	19.6 (33)
Adequacy of MDD-W Food groups consumed by the household in last 7days	Inadequate <5 groups	38.0 (74)	41.1 (69)
	Adequate > = <5 groups	62.0 (121)	58.9 (99)
Mean (SD) MDD-W Food groups consumed by the household in last 24h		3.3 (1.4)	3.5 (1.6)
Mean (SD) MDD-W Food groups consumed by the household in last 7 days		4.9 (1.8)	5.3 (2.2)
